# The Role of Matrix Metalloproteinases in Colorectal Cancer

**DOI:** 10.3390/cancers6010366

**Published:** 2014-02-10

**Authors:** Anan H. Said, Jean-Pierre Raufman, Guofeng Xie

**Affiliations:** Division of Gastroenterology and Hepatology, Veterans Affairs Maryland Health Care System, University of Maryland School of Medicine, Baltimore, MD 21201, USA; E-Mails: anansaid@umaryland.edu (A.H.S.); jraufman@medicine.umaryland.edu (J.-P.R.)

**Keywords:** matrix metalloproteinases (MMPs), colorectal cancer, tissue inhibitors of metalloproteinases (TIMPs), matrix metalloproteinase inhibitors (MMPIs)

## Abstract

In the United States, colorectal cancer (CRC) is the third leading cause of cancer mortality, with limited treatment options for those with advanced disease. Matrix metalloproteinases (MMPs) are important for maintaining extracellular homeostasis but also play a prominent role in cancer cell invasion and dissemination. Expression levels of MMP-1, -2, -7, -9 and -13 correlate with worse outcomes; MMP-12 expression appears to be protective. Hence, MMPs are attractive therapeutic targets. Previous clinical trials using broad-spectrum MMP inhibitors were disappointing because of off-target toxicity and lack of efficacy. Now, the availability of safer, more selective inhibitors has renewed interest in therapeutic targeting of MMPs.

## 1. Introduction

In the United States, colorectal cancer (CRC) is the third leading cause of cancer and cancer death in both men and women. In 2013, ~142,820 new cases of CRC are anticipated and ~50,830 of these are likely to result in premature death [1]. In the U.S., the lifetime risk of developing CRC is estimated at 1 in 20; those with CRC have an overall 5-year survival rate of ~65%, primarily dependent on pathological stage at diagnosis [2]. The ~40% of cases diagnosed with disease limited to the colon have greater than 90% 5-year survival rate. Five-year survival decreases to ~70% with regional spread, and for the 20% of CRC diagnosed with distant metastases, the 5-year survival rate drops to 12.5% [2]. While surgical resection of localized cancer is the mainstay of treatment, those with advanced metastatic disease have few therapeutic options. Thus, there is great interest in expanding our understanding of the molecular mechanisms that underlie colon cancer dissemination, and using this information to develop novel therapeutic strategies.

CRC pathogenesis results from a complex, multistage process progressing from neoplastic transformation of normal cells, tissue invasion, vascular intra- and extravasation, and ultimately seeding in another organ, usually the liver. In all tissues, the extracellular matrix (ECM) provides a structural and biochemical framework for cell support and scaffolding, with a range of functions important for regulating both inter- and intra-cellular signaling, and for cellular differentiation, adhesion and invasion. Cancer cells interact with the ECM, and structural remodeling is important for migration from a primary tumor site. Proteins comprising the ECM play critical roles in cell proliferation and migration, and different proteases control ECM remodeling and degradation. One specific group of proteolytic enzymes, matrix metalloproteinases (MMPs), were studied extensively as key mediators of ECM degradation and in the processing of other bioactive molecules [3]. MMPs also regulate cell surface growth factor “shedding” which regulates the proteolytic release of several proteins such as growth factors, chemokines and adhesion molecules. In a variety of different cancers, increased MMP expression and activation generally promote hallmarks of tumor progression including angiogenesis, invasion and metastasis, and correlate with shortened survival. Nonetheless, more recently, some MMPs were shown to have tumor protective effects [4].

MMPs comprise a large family of at least 25 zinc-dependent endopeptidases capable of degrading all components of the ECM and are categorized primarily by their structural features as gelatinases, collagenases, membrane-type, stromelysins and matrilysins. MMPs have a common domain structure including a pro-peptide, a catalytic domain, a hemopexin-like C terminal domain, and a hinge region that links the catalytic site with the hemopexin domain [5]. They are synthesized as secreted or membrane-associated inactive zymogens, and must be proteolytically processed to an active state. This processing involves removal of a cysteine residue that interacts with zinc ions from the active site, thereby resulting in MMP activation.

## 2. Specific Roles of MMPs in CRC

### 2.1. MMP-1 and MMP-13 (Collagenases)

MMP-1 is a collagenase that degrades ECM, specifically targeting type I, II and III collagens, the major components of the interstitial stroma. In CRC, MMP-1 expression correlates with advanced colon cancer stage and poor prognosis [6]. In a study involving 142 human specimens, the level of invasion, lymph node involvement and metastasis were all associated with elevated levels of MMP-1 [7]. A subsequent study revealed that MMP-1 expression was significantly different in primary and metastatic CRC, with strong MMP-1 expression seen in primary cancers with lymph node involvement, but decreasing MMP-1 expression observed in metachronous metastases [8]. MMP-1 was not over-expressed in synchronous metastases at time of diagnosis, suggesting that MMP-1 plays a role in initial stages of invasion but is less critical once metastases are established.

To identify the role of MMP-1 in colon cancer metastasis, a set of experiments were designed to study its function in “tumor self-seeding”, wherein circulating tumor cells colonize the tumor of origin, allowing its enrichment with more aggressive cells. In addition to other tumor-derived cytokines that can act as circulating tumor cell attractants, MMP-1 was shown to be a key mediator of such primary tumor invasion [9]. In two different colon cancer cell lines, muscarinic receptor activation was also shown to stimulate ~50-fold increase in MMP-1 mRNA expression compared to control [10]. Colon cancer cell invasion and migration correlated with increased MMP-1 gene expression, and these actions were reversed by chemical inhibitors or neutralizing antibodies against MMP-1 [11].

MMP-1 genetic polymorphisms are also associated with increased CRC susceptibility. A guanine insertion/deletion polymorphism was identified in the promoter region of MMP-1 wherein one allele has a single guanine (1G) nucleotide and another has two guanine (2G) nucleotides [12]. This 2G nucleotide allele has increased transcriptional activity compared to the 1G nucleotide allele, and was associated with increased metastasis and susceptibility to CRC.

MMP-13, another member of the collagenase sub-family of MMPs, shares structural homology with MMP-1, although MMP-13 was shown to be most effective in degrading type II collagen. Previous studies showed that increased MMP-13 immunohistochemical staining correlated with diminished survival [13]. A potential role for MMP-13 as a marker for CRC was studied, revealing increased MMP-13 expression with advanced cancer stage and nearly eight-fold increased risk of post-operative relapse compared to those without MMP-13 overexpression [14].

### 2.2. MMP-2 and MMP-9 (Gelatinases)

MMP-2 and MMP-9 comprise the gelatinase sub-family of MMPs, and although the main substrates for these enzymes are type IV collagen and gelatin, they also share proteolytic activity against other extracellular matrix molecules. In CRC, many studies reveal a correlation between increased MMP-2 and MMP-9 expression and worse outcome. Increased plasma MMP-2 expression was observed in lymph node-positive patients with CRC compared to those without lymph node metastasis [15].

Several studies examined the utility of serum MMPs as markers for CRC invasion. One study found that MMP-2 and -9 protein levels were expressed at significantly higher ratios in the sera of persons with CRC compared to normal controls. This finding had greater diagnostic sensitivity than two other biomarkers currently used in clinical practice, CEA and CA19-9 [16]. In T3-T4 node-negative CRC, MMP-9 over-expression, CEA greater than 5 ng/mL and lymphatic invasion were associated with poor prognosis.

Cancer cells require integrins for adhesion and MMPs for proteolysis. Kryczka *et al*. examined the interaction of β1 integrins with MMP-2 in colon cancer cells. These studies revealed up-regulation of MMP-2 expression in invasive CRC; MMP-2 degrades β1 integrins, thereby enhancing motility and decreasing cell adhesion [17].

Multiple signaling pathways play a role in the activation of gelatinases. Smad proteins are involved in TGF-β signaling and function in cell cycle regulation, differentiation and apoptosis. Smad4 binds to receptor-regulated SMADs, and suppresses colon cancer cell migration by regulating MMP-9 activity. In colon cancer, over-expression of p38 gamma MAPK was shown to lead to increased c-Jun synthesis, resulting in enhanced MMP-9 transcription and MMP-9-dependent invasion [18]. Also, TGF-β receptor kinase inhibitors reduce expression of MMP-9 and block CRC metastasis to the liver [19,20].

In animal models and humans with inflammatory bowel disease, which is commonly associated with progression to colitis-associated colon cancer (CAC), MMP-9 is highly expressed in inflamed intestine. Compared to WT mice, MMP-9-null mice exhibited increased susceptibility to CAC with increased Notch-1 activation, an important transcription factor in determining epithelial lineage, as well as a reciprocal decrease in β-catenin expression, a proto-oncogene with a central role in regulating both gene transcription and Wnt signaling [21]. Therefore, although MMP-9 mediates pro-inflammatory responses in colitis, it has a protective role and acts as a tumor suppressor in CAC.

### 2.3. MMP-7 (Matrilysin)

Over-expression of MMP-7 is seen in ~80% of CRC [22]. Serum levels of MMP-7 are associated with decreased survival in advanced CRC and correlate with cancer progression [23]. Using a fluorescent MMP-7 probe to detect colorectal adenomas in adenomatous polyposis coli (*Apc*)^+/Min-FCCC^ mice that spontaneously develop colorectal adenomas, more than 92% of colon adenomas were identified, suggesting its potential use for early detection and intervention [24].

MMP-7 promotes cancer invasion via proteolytic cleavage of ECM proteins and also activates other MMPs, including proMMP-2 and proMMP-9 to promote cancer cell invasion [25]. This enzyme also modifies non-ECM proteins, resulting in their activation, degradation and proteolytic shedding. Through proteolytic shedding of the ectodomain of proHB-EGF to form the mature HB-EGF and activation of EGFR signaling, MMP-7 is involved in regulating cell proliferation and apoptosis [25]. Furthermore, in human colon cancer cell lines, activation of muscarinic receptors results in substantial increases in MMP-7 expression [10].

In SCID mice, inoculation with human colon cancer cells that overexpress MMP-7 increases tumor invasion and metastasis [26]. In conformity with its role in metastasis, down-regulating MMP-7 with antisense oligonucleotides inhibited liver metastasis [27]. Additionally, in the *Min* (multiple intestinal neoplasia) mouse model, gene knockout of MMP-7 significantly reduced colon cancer multiplicity and tumor size, thereby highlighting the role of MMP-7 in tumorigenesis [28].

### 2.4. MMP-12 (Metalloelastase)

MMP-12, also called metalloelastase, is not categorized in any of the MMP subfamilies, and is known to degrade many different substrates. Whereas it is predominantly expressed in macrophages, in several studies MMP-12 was protective in CRC; its inhibition was found to be potentially deleterious [29,30]. Although increased expression of MMP-12 was found in CRC, expression levels were noted to be higher in primary tumors associated with no hepatic metastasis compared to those associated with liver metastasis [31]. In particular, MMP-12 expression was observed to decrease VEGF (vascular endothelial growth factor) expression, as well as to cause an increase in angiostatin, an endogenous angiogenesis inhibitor [32]. In line with these findings, several studies reported MMP-12 expression to be associated with both reduced tumor growth and increased overall survival [33,34].

## 3. Tissue Inhibitors of Metalloproteinases

Tissue inhibitors of metalloproteinases (TIMPs) comprise a family of four homologous protease inhibitors (TIMP 1-4) that are naturally-occurring specific endogenous inhibitors of metalloproteinases that minimize ECM degradation. By forming complexes with most MMPs, TIMPs inhibit their proteolytic activity. TIMPs are involved in many biological activities including migration, invasion, cell proliferation, angiogenesis and apoptosis [35]. Dual activities of TIMP-1 have been observed—these molecules play a role in controlling biological actions of MMPs as well as functioning independently of MMP activity. In human colon cancer cells, TIMP-1 conferred resistance against cytotoxicity caused by TNF-α and IL-2, and contributed to clonogenicity and tumor growth during early tumor formation [36]. However during later stages of tumor growth, aberrant glycosylation of TIMP-1 was observed with resulting loss of TIMP-1 inhibition of collagenases, fostering more invasive tumors. TIMP-1 inhibits MMP-1, -3, -7 and - 9 preferentially [35]. Increased levels of TIMP-1 were observed in patients with colon cancer, which also was associated with a worse outcome [37]. In a study examining potential biomarkers for early CRC detection, total levels of plasma TIMP-1 identified persons with CRC with high sensitivity and specificity, and had even higher predictive value for right-sided colon cancer [38].

TIMP-2 inhibits gelatinases, MMP-2 and MMP-9, and also serves as an adaptor protein for pro-MMP-2 activation [39]. In Korean CRC patients, a genetic polymorphism in TIMP-2 was associated with increased risk of metastasis and worse prognosis [40]. Colon cancer cells with siRNA knockdown of CD133, a putative stem cell and cancer stem cell marker, displayed down-regulated TIMP-2 expression and decreased invasiveness [41].

In addition to inhibiting MMPs, TIMP-3 also inhibits a family of peptidase proteins similar to MMPs named ADAMs (a disintegrin and metalloproteinase). In colon cancer cell lines, TIMP-3 suppresses neoplasia by inducing apoptosis, an action thought to be mediated by stabilization of TNF-α receptors. TIMP-3 was reduced in the stroma surrounding invasive CRC [42]. In a study examining the use of TIMP-3 as biotherapy for CRC, adenovirus-mediated TIMP-3 transduction arrested cancer cell growth and induced apoptosis. *In vitro* data also revealed that increasing TIMP-3 levels reduced adhesion, migration and invasiveness of a human colon cancer cell line, while *in vivo* studies revealed that TIMP-3 transduction reduces both tumor growth and liver metastasis [43].

TIMP-4 is an inhibitor of MMP-2 catalytic activity; strong cytoplasmic staining of TIMP-4 in rectal cancer tissues predicted longer survival [44]. In the same study, multivariate analysis revealed that stromal cytoplasmic staining for TIMP-3 was the only marker of independent prognostic value in CRC, thereby highlighting its role as a potential biomarker for CRC. Table 1 summarizes the prominent MMPs and TIMPs and their roles in colorectal cancer.

## 4. Targeting MMPs in Cancer Therapy and Future Directions

Given current knowledge about MMPs and their correlation with neoplasia, molecular inhibitors of MMPs (MMPIs) were developed and tested in clinical trials. In the early 1990s, MMPIs developed to target cancer showed promise in phase I and II trials, and indeed showed inhibitory effects on growth of both primary and metastatic CRC. However, many phase III trials ended without revealing survival advantages and the use of these agents was associated with substantial toxicity. Synthetic MMPIs were thought to have failed because they lacked selectivity; these agents may have blocked the activity of MMPs that were not over-expressed in a particular cancer or blocked the activity of MMPs with tumor suppressor properties [45].

**Table 1:** Prominent MMPs and TIMPs in colorectal cancer (CRC).

MMP/TIMP nomenclature	Actions	Role in CRC	References
MMP-1	Collagenase-1	Expression correlates with CRC invasion and metastasis	[6,7,8]
MMP-2	Gelatinase A	Expression correlates with CRC invasion	[15,16,17]
MMP-7	Matrilysin	Expression correlates with CRC cell proliferation, invasion and metastasis	[25,26,27,28]
MMP-9	Gelatinase B	Expression correlates with CRC metastasis and is protective in colitis-associated colon cancer	[18,19,20,21]
MMP-12	Metalloelastase	Expression correlates with reduced CRC growth and increased survival	[31,32,33,34]
MMP-13	Collagenase-3	Expression correlates with diminished CRC survival	[13]
TIMP-1	Inhibits most MMPs	Expression correlates with right-sided CRC and poor survival	[37,38]
TIMP-2	Inhibits MMP-2 and MMP-9	Reduced expression correlates with CRC invasion and worse prognosis	[40,41]
TIMP-3	Inhibits MMPs and ADAMs	Decreased expression correlates with increased CRC invasion	[42]
TIMP-4	Inhibits MMP-2	Expression in rectal cancer correlates with longer survival	[44]

Another potential reason for the therapeutic failure of MMPIs in these earlier trials was that despite animal studies showing greatest efficacy in early-stage disease, research subjects were enrolled late in their disease course, when they already had metastatic cancer. This point is highlighted by the results of a study involving a synthetic MMPI, MMI270, in a rat model of colon cancer metastasis [46]. MMI270 competitively binds to zinc in the active state of several MMPs including MMP-1, -2, -3, -9 and -13 resulting in their inhibition. Early administration of MMI270, immediately after removing primary colon cancers, significantly reduced lung metastasis compared to delayed administration.

In the hope of increasing their substrate selectivity, newer structure-based inhibitors of MMPs were developed that take into account the three-dimensional conformation of the enzymatic active site, rather than the previous substrate-based broad-spectrum MMPIs. Other inhibitors of MMPs include SB-3CT which covalently binds to the MMP-2 active site to return the enzyme to its pro-peptide state [47]. An additional group of MMPIs under investigation are chemically-modified tetracyclines (CMTs). Although they lack intrinsic antibiotic activity, their mode of action is thought to involve binding of zinc or calcium ions, or through transcriptional regulation of MMP expression [47]. Another method of targeting MMPs is through microRNAs (miRNAs) that function in transcriptional and post-transcriptional gene regulation. Several of these biological regulators inhibit secretion of MMPs and block MMP activity, thus highlighting their potential as tumor suppressors [4]. One such example was noted with use of antisense oligonucleotides targeting MMP-7 mRNA, which prevented human colon cancer cell invasion *in vitro* by inhibiting expression of MMP-7 [27]. In relation with this study, using antisense oligonucleotides to MMP-7 mRNA in human colon cancer cell line xenograft models showed inhibition of basement membrane penetration, and suppression of liver metastases [48]. To further highlight the emerging role of targeting microRNAs in colon cancer, a recent set of experiments were designed to examine the role of miRNA-34a (miR-34a) in human colon cancer. This miRNA has been shown to be a transcriptional target of p53, a gene that is important in regulating the cell cycle and functions as a tumor suppressor. By transfecting miR-34a into human colon cancer cells, the expression of both MMP-1 and MMP-9 was decreased substantially and ultimately led to inhibition of human colon cancer cell migration and invasion [49].

Although therapeutic inhibition of MMPs previously encountered difficulties, active studies are underway to identify how best to target MMPs in CRC. The role of MMPs as biomarkers of CRC, their use in monitoring therapeutic responses and to identify persons who will likely respond best to a particular chemotherapeutic regimen are all current avenues of investigation. Taken together, based on the role and functions that MMPs play in a host of pathological conditions extending well beyond cancer, the ultimate goal of future work is to develop effective therapy by selective targeting of MMPs.
